# Echocardiographic assessment of maximum and minimum left atrial volumes: a population-based study of middle-aged and older subjects without apparent cardiovascular disease

**DOI:** 10.1007/s10554-014-0533-6

**Published:** 2014-09-12

**Authors:** Egil Henriksen, Jonas Selmeryd, Jerzy Leppert, Pär Hedberg

**Affiliations:** 1 Department of Clinical Physiology, Västmanland County Hospital, SE-72189 Västerås, Sweden; 2 Centre for Clinical Research, Västmanland County Hospital, Uppsala University, Västerås, Sweden

**Keywords:** Echocardiography, Left atrium, General population, Reference values

## Abstract

The aim of the present study was to obtain reference values of maximum and minimum left atrial volumes (maxLAV and minLAV, respectively) in a population-based subset without apparent cardiovascular disease or other factors potentially associated with left atrial enlargement. Because left ventricular diastolic dysfunction is commonly found in elderly subjects, we also tried to identify the presence of possible preclinical diastolic dysfunction in the study population. A population-based sample of 168 subjects (127 men and 41 women) underwent two-dimensional echocardiography using the single-plane disc method to determine maxLAV and minLAV. maxLAV and minLAV were indexed to body surface area (maxLAVi and minLAVi, respectively). maxLAVi was independent of age and sex, and produced reference limits (mean ± 1.96 SD) of 15–37 mL/m^2^. minLAVi was correlated with age, and produced estimated reference limits of 3–15 and 7–23 mL/m^2^ in 40- and 80-year-old subjects, respectively. Based on the age-dependent reference values from the European Association of Cardiovascular Imaging, <5 % of the study population had possible preclinical left ventricular diastolic dysfunction. The present study established normal ranges for maxLAVi and minLAVi in a well-characterized population-based subset without apparent cardiovascular disease or other factors potentially associated with left atrial volume enlargement.

## Introduction

For decades, the left ventricular ejection fraction has been used to predict cardiovascular outcomes. More recent data have shown that maximum left atrial volume (maxLAV) indexed for body surface area (maxLAVi) is an independent predictor of survival after myocardial infarction and congestive heart failure [1–5]. It has also been suggested that maxLAVi is less liable to fluctuate with short-term changes in loading conditions compared with Doppler filling indices and, therefore, maxLAVi has been proposed as a more specific marker of a persistent increase in left ventricular (LV) filling pressure [6]. The fact that maxLAVi carries independent prognostic information and seems to add indispensable information to the Doppler filling indices for assessing LV filling highlights the importance of measuring maxLAVi. Far less attention has been paid to the possibility that elevated filling pressure may cause a reduction in left atrial (LA) contractility, with a corresponding enlargement of minimum left atrial volume (minLAV). Consequently, minLAV indexed for body surface area (minLAVi) may have an additive value to the maxLAVi and Doppler filling indices in the evaluation of LV diastolic function [7].

In the guidelines of the American Society of Echocardiography/European Association of Cardiovascular Imaging (ASE/EACVI) [8], the given upper limit of maxLAVi is based on small cross-sectional studies performed in relatively young patient-based subsets during the 1980s [9, 10], in a small subgroup of 44 young patients [11], and in a group of 92 healthy volunteers [12]. In our clinical experience, the ASE/EACVI guidelines’ recommended upper reference limit for maxLAVi is frequently encountered in adults without known or apparent cardiovascular disease. We set out to examine maxLAVi and minLAVi in a well-characterized population-based sample of people without apparent cardiovascular disease or other factors potentially associated with LA enlargement.

## Method

### Study population

The participants were recruited from the Västmanland Myocardial Infarction Study (VaMIS). Consecutive patients hospitalized for acute myocardial infarction from November 2005 to May 2011 were included in the VaMIS study. For each patient included, a control subject was recruited from the general population. A person with the nearest date of birth, same sex, and living in the same municipality as the VaMIS patient was identified in the Swedish Population register, in which all Swedish citizens are registered. All subjects underwent clinical examination, electrocardiography (ECG), echocardiographic examination, and blood sampling. From the control group of the VaMIS study (*n* = 855), we excluded individuals with a history of diabetes mellitus, hypertension, non-sinus rhythm, myocardial infarction, angina pectoris, transient ischemic attack/stroke, and symptoms of peripheral artery disease, as well as those taking regular cardiovascular or antihypertensive medication. In addition, subjects with a blood pressure ≥140/90 mmHg measured at two separate occasions, a body mass index (BMI) >35, or categorized as New York Heart Association class II–IV were excluded from the study. Individuals with an abnormal echocardiographic wall motion score index, significant valvular disease, or missing values were also excluded. Finally, 168 individuals (127 men and 41 women) without apparent cardiovascular disease or comorbidity known to be associated with LV filling disorders were included in the analyses.

The study was approved by the Ethics Committee of Uppsala University, Sweden (Dnr 2005:382). All participants gave their written informed consent.

### Image acquisition

A two-dimensional (2D) echocardiographic examination dedicated for research was performed using a commercially available Vingmed Vivid Seven (General Electric, Horten, Norway). All examinations were performed by an experienced echocardiographer (P.H.). The images were obtained in the left lateral recumbent position using a phased array transducer in the standard parasternal and apical views. The ECG-triggered 2D images and Doppler data were stored digitally in a cine loop format. Three consecutive cardiac cycles were recorded during quiet breathing.

### Echocardiographic analysis

The analysis was performed by one of two experienced physicians (J.S. and P.H.) at least 3 months after the image acquisition and was performed using commercially available software (Echo PAC, PC version 110, Horten, Norway) with anonymized images. The LV cavity and wall dimensions were measured from the 2D images using the leading edge to leading edge principle. LV mass was estimated using the ASE-recommended formula [8].

In the assessment of LA volumes, the single-plane disk method was used in the apical 4-chamber view. The stored loops of this view were dedicated to LA visualization and oriented to maximize the LA area. maxLAV (i.e., end systolic) assessment was performed using the frame immediately preceding the mitral valve opening, and minLAV (i.e., end diastolic) was obtained using the frame contiguous to mitral valve closure. The LA endocardial border, excluding the LA appendage and the confluences of pulmonary veins, was traced with a straight line connecting the septal and lateral mitral leaflet base attachment points to the annulus as the superior border of the outlined area.

### Doppler filling indices

Mitral inflow was recorded using pulsed Doppler at the tips of the mitral leaflets. The peak early (E) and late (A) transmitral diastolic flow velocities, the E/A ratio, and the deceleration time of the early filling velocity (MV-Edt) were obtained. The peak velocity of the early diastolic wave (TD-e′) was measured using pulsed-wave tissue Doppler with the sample volume close to the mitral valve annulus in the apical 4-chamber view in the septal (TD-e′ septal) and lateral (TD-e′ lateral) walls. The E/e′ ratio was calculated based on the transmitral E wave and the average of TD-e′ lateral and TD-e′ septal (TD-e′ mean).

Because LV diastolic dysfunction is frequently observed in aged people and is associated with LA enlargement, we made an effort to evaluate the presence of possible preclinical diastolic dysfunction in the study population. Based on an algorithm and age-related reference values presented by the EACVI [13], possible diastolic dysfunction grade I was defined as the combination of TD-e′ mean <9 cm/s, E/A ratio <0.80, and MV-Edt >200 ms in subjects aged ≤60 years. The corresponding cutoffs for subjects aged >60 years were TD-e′ mean <6 cm/s, E/A ratio <0.60, and MV-Edt >200 ms. Possible diastolic dysfunction grade II–III was defined as the combination of TD-e′ mean <9 cm/s, E/A ratio ≥0.80, and E/e′ ratio ≥9 in participants aged ≤60 years. In subjects aged >60 years, the corresponding values were TD-e′ mean <6 cm/s, E/A ratio ≥0.60, and E/e′ ratio ≥9.

### Intra- and interobserver reproducibility of LA volume measurements

The reproducibility of LA volume measurement was determined by two sets of measurements in two separate readings in a sample of 19 randomly selected subjects. To determine the intraobserver reproducibility, the acquisitions were reanalyzed by the original observer at least 3 months after the first evaluation. The interobserver reproducibility was tested by a second reader using the same 19 participants.

### Statistical analysis

Continuous data are expressed as the mean ± standard deviation (SD) and categorical data are expressed as absolute values and percentages. The mean values for continuous variables were compared using the *t* test. Adjusted means were estimated and compared by analysis of covariance. Categorical variables were compared using Fisher’s exact test. Univariate relationships are expressed as Pearson´s correlation coefficients. Reference limits were estimated as the mean value ± 1.96 SD. In cases of age dependency (minLAVi), a parametric derivation of age-related reference limits was performed according to Altman [14], and Royston and Wright [15]. A least-square regression analysis was used to model the mean of minLAVi as a function of age. Subsequently, the scaled absolute residuals (i.e., absolute residuals multiplied by the square root of π/2) of the regression of minLAVi on age was used to model the function of SD on age. If the scaled absolute residuals showed no trend with age, the SD was estimated as the SD of the unscaled original residuals. Bivariate scatter plots with running-line smoothers were used to confirm the appropriateness of the linear models. Goodness of fit was evaluated via residual density plots, normal probability (P–P) plots, quantile (Q–Q) plots, and residuals-versus-fitted values plots. Centile curves were estimated using the formula: centile = mean + K × SD, where K is the corresponding centile of the standard Gaussian distribution (in our case, the 2.5th and 97.5th centile curves were estimated with K = ± 1.96). STATA version 12.1 (StataCorp LP, College Station, TX, USA) was used for all analyses. A *P* value <0.05 was considered significant.

## Results

### Baseline characteristics

The baseline characteristics of the participants are presented in Table 1. The mean age ± SD was 63.3 ± 9.5 (range 45–81) years and 59.9 ± 9.7 (range 38–81) years for women and men, respectively. The measured values of LV diastolic function were within the normal age-adjusted intervals in the vast majority of the participants. An E/e′ ratio of ≥9 was found in 5.4 % of the study population, and no individual had an E/e′ ratio >14. Possible preclinical diastolic dysfunction was present in eight (4.8 %) of the participants, three of whom had an E/e′ ratio of ≥9. An N-terminal pro-brain natriuretic peptide (NT-proBNP) level >200 ng/L was found in 7 % of the subjects. Two participants had an NT-proBNP level >300 ng/L (546 and 548 ng/L, respectively). They were both men, aged 59 and 69 years, with E/A ratios of 0.91 and 1.34, respectively, and E/e′ ratios of 4.2 and 5.3, respectively.

**Table 1:** Characteristics of the study population (*n* = 168)

	Men *n* = 127	Women *n* = 41
Age (years)	59.9 ± 9.7	63.3 ± 9.5
Current smoker	14 (11)	5 (12)
Height (cm)	179 ± 6	163 ± 6
Weight (kg)	82 ± 11	66 ± 11
Body mass index (kg/m^2^)	25.5 ± 2.7	25.0 ± 3.4
Body surface area (m^2^)	2.02 ± 0.15	1.73 ± 0.17
Systolic blood pressure (mmHg)	132 ± 8	128 ± 12
Diastolic blood pressure (mmHg)	79 ± 7	76 ± 7
NT-proBNP (ng/L)	69 ± 81	102 ± 61
Echocardiography		
LV end diastolic diameter (mm)	50.2 ± 4.1	46.5 ± 3.9
Interventricular septum (mm)	10.5 ± 1.5	9.5 ± 1.4
LV posterior wall (mm)	9.5 ± 1.1	8.7 ± 1.2
LV mass (g)	189 ± 38	148 ± 31
MV-E (cm/s)	53.9 ± 11.6	60.9 ± 11.2
MV-A (cm/s)	47.9 ± 11.4	54.5 ± 11.7
E/A ratio	1.19 ± 0.41	1.17 ± 0.32
MV-Edt (ms)	232 ± 62	211 ± 46
TD-e′ mean (cm/s)	9.1 ± 2.0	8.9 ± 2.2
E/e′ ratio	6.1 ± 1.4	7.1 ± 1.8
Possible DD, grade I	4 (3)	1 (2)
Possible DD, grade II–III	1 (1)	2 (5)

*NT*-*proBNP* N-terminal pro-brain natriuretic peptide, *LV* left ventricular, *MV*-*E* mitral valve E-wave, *MV*-*A* mitral valve A-wave, *MV*-*Edt* mitral valve E-wave deceleration time, *TD*-*e′ mean* peak early diastolic mitral annular velocity (average of septal and lateral walls), *DD* diastolic dysfunction

The data are presented as the mean ± standard deviation or number (percentages)

### LA volumes

The LA volumes in men and women are presented in Table 2. maxLAV was significantly greater in men compared with women; however this sex difference disappeared after indexing maxLAV to body surface area. Although minLAV did not differ between women and men, after indexing to body surface area, there was a trend toward a higher minLAVi in women compared with men (12.0 vs 10.7 mL/m^2^; *P* = 0.056). However, after adjusting for age, this sex difference in minLAVi was attenuated (estimated mean, 11.6 vs 10.8 mL/m^2^ for women and men, respectively; *F* = 1.54, *P* = 0.22).

**Table 2:** Maximum and minimum left atrial volumes according to sex

LA volumes	Men *n* = 127	Women *n* = 41	*P* value^a^
	Mean ± SD	Mean ± SD	
maxLAV (mL)	51.7 ± 11.9	43.8 ± 10.6	<0.001
minLAV (mL)	21.4 ± 7.5	20.9 ± 7.5	0.71
Indexed to BSA			
maxLAVi (mL/m^2^)	25.6 ± 5.9	25.2 ± 5.3	0.69
minLAVi (mL/m^2^)	10.7 ± 3.9	12.0 ± 3.9	0.056

*maxLAV* maximum left atrial volume, *minLAV* minimum left atrial volume, *BSA* body surface area

^a^For differences between men and women (*t* test)

Table 3 shows the univariate analysis of the relationships between demographic and anthropometric measures and maxLAVi and minLAVi. There were no significant relationships between maxLAVi and age, sex, or measures of body size. In contrast, minLAVi was significantly correlated with age (although the correlation coefficient was low) and borderline significantly correlated with sex. Even after indexing for body surface area, minLAVi remained significantly associated with height and weight. However, in multivariable linear regression models that included age, sex, and height, weight, or BMI entered separately, only age remained a significant independent predictor of minLAVi (*P* < 0.001 in all models).

**Table 3:** Univariate correlations between demographic and anthropometric measures and maximum and minimum left atrial volumes indexed for body surface area

	maxLAVi		minLAVi	
	*r*	*P* value	*r*	*P* value
Sex	0.03	0.69	–0.15	0.056
Age	0.11	0.17	0.39	<0.001
Height	–0.01	0.87	–0.20	0.011
Weight	–0.05	0.56	–0.19	0.016
BMI	–0.05	0.54	–0.07	0.37

*maxLAVi*, maximum left atrial volume indexed for body surface area; *minLAVi*, minimum left atrial volume indexed for body surface area; *BMI*, body mass index

The reference interval for maxLAVi was estimated as 15–37 mL/m^2^ (mean ± 1.96 SD). As expected, 2.4 % (*n* = 4) of the participants had a maxLAVi > 37 mL/m^2^.

**Fig. 1:**
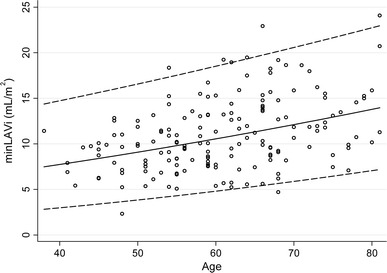
Minimum left atrial volume indexed for body surface area (minLAVi) according to age, and reference curves for estimated mean (*solid line*) and −1.96 SD and +1.96 SD (*dashed lines*)

Because of the significant relationship between minLAVi and age, we estimated the reference interval via general linear regression using minLAVi as the dependent variable and age as the predictor. A slight departure from a Gaussian distribution and some heteroskedasticity of the residuals were effectively corrected by an initial square root transformation of minLAVi. The final back-transformed model of estimation of the reference interval for minLAVi was (1.85 + 0.023 × age)^2^ ± (1.96 × 0.54)^2^ and is displayed graphically in Fig. 1. The reference intervals for maxLAVi and minLAVi are presented in Table 4.

**Table 4:** Reference intervals for maximum and minimum left atrial volumes indexed for body surface area

	Reference interval^a^
maxLAVi (mL/m^2^)	15–37
minLAVi (mL/m^2^)	
At 40 years of age	3–15
At 60 years of age	5–18
At 80 years of age	7–23

*maxLAVi*, maximum left atrial volume indexed for body surface area; *minLAVi*, minimum left atrial volume indexed for body surface area

^a^Reference interval for maxLAVi was estimated as the mean ± 1.96 SD. Reference intervals for minLAVi were calculated according to the regression equation: (1.85 + 0.023 × age ± 1.96 × 0.54)^2^

### Reproducibility of LA volume measurements

The intra- and interobserver reproducibility, expressed as the absolute mean difference ± SD, and the coefficient of variation (CV) for the maxLAV measurements were 2.6 ± 4.1 mL (CV 8.0 %) and 1.2 ± 6.1 mL (CV 11.9 %), respectively. The corresponding figures for the intra- and interobserver reproducibility of the minLAV measurements were 1.3 ± 3.8 mL (CV 16.1 %) and 1.6 ± 4.5 mL (CV 19.2 %), respectively.

## Discussion

In the present population-based study, the distributions of maximum and minimum LA volumes were studied in 168 randomly selected subjects without apparent cardiovascular disease or comorbidity known to be related to LA enlargement. The current data confirm that maxLAVi was independent of age and sex, and displayed an upper normal limit of 37 mL/m^2^. In contrast, minLAVi was correlated with age, and exhibited estimated upper reference limits of 15 mL/m^2^ and 23 mL/m^2^ in subjects aged 40 and 80 years, respectively.

LA enlargement has been proposed as a barometer of diastolic burden and as a predictor of stroke, heart failure, and cardiovascular death. Therefore, it is important to include maxLAVi in the standard echocardiographic examination [1–6, 16]. However, this apparently simple assignment presents several problems. Several imaging techniques, such as echocardiography, cardiomagnetic resonance imaging, and high-resolution computed tomography, have been used to obtain LA volumes [10, 17–21]. The geometric models and methods used for LA measurement vary between single or biplane methods, 3D models, area-length models, and disc models. The differences in LA volume measurements between different studies and methods are disturbingly large and emphasize the need for proper population-based reference values for the actual method used.

In the present study, the upper limit of the reference interval for maxLAVi was 37 mL/m^2^, as measured using the apical 4-chamber single-plane disk method. In comparison, the mean + 2 SD of biplane Simpson indexed LA volumes reported in the 1980s by two smaller studies (*n* < 60) of young healthy volunteers (mean age <40 years) were slightly lower, at 34 and 32 mL/m^2^, respectively [9, 10]. In a healthy subgroup (n = 44) with a mean age of 39 years and free from possible diastolic dysfunction, Tsang et al. [11] reported an upper reference limit (mean + 2 SD) of 32 mL/m^2^. In a patient population free from apparent cardiovascular disease, Thomas et al. [12] observed upper reference limits of 39 and 35 mL/m^2^ in the subgroups younger (*n* = 47) and older (*n* = 45) than 50 years, respectively. Identical to our finding, Kou et al. [22] reported an upper reference limit of 37 mL/m^2^ in a very recent large multicenter study of healthy volunteers (*n* = 734). Interestingly, their estimated upper reference limit of maxLAVi from the single-plane 4-chamber view was exactly the same (i.e., 37 mL/m^2^) as their biplane estimate.

In the ASE/EACVI guidelines, the upper limit of maxLAVi is reported as ≤28 mL/m^2^ [8]. Obviously, the recommendation is not only derived from cross-sectional studies of healthy subjects, but is also based on the estimated risk related to LA volume and on expert opinion. According to previous cross-sectional studies, the guideline-recommended reference limit of 28 mL/m^2^ would approximately correspond to a population mean + 1 SD in an apparently healthy population. The use of 1 SD to define normality is notable, as it surely will categorize otherwise-healthy individuals as diseased. Certainly, LA volumes ≥28 mL/m^2^ have been reported to be predictive of future congestive heart failure [4] and compound cardiovascular events [23]. An even lower LA volume of ≥27 mL/m^2^ has been reported as being predictive of future atrial fibrillation in patients with hypertrophic cardiomyopathy [24]. Most biological risk markers measured on a continuous scale are likely to display a risk continuum without an exact point above which the risk suddenly increases, and an increased risk can even be detected at levels that are considered to be within the distribution of the healthy population (e.g., serum cholesterol [25] and blood pressure [26] ). In fact, the concept of “optimal” prognostic thresholds has recently been questioned [27]. Risk stratification is not straightforward, as it requires multifactorial considerations in a clinical context and cannot be simplified axiomatically into a threshold of a single parameter. In our opinion, the reference limits should be thresholds dedicated to inform the clinician about the distribution of a marker in the healthy population, separate from prognostic or therapeutic decision limits.

As expected, the maxLAV was larger in men than in women in the present study. However, this sex difference disappeared after adjusting for body surface area. Some studies have not found a relationship between maxLAVi and age [10, 12, 19], whereas others have done so [28]. The present data showed no significant correlation between age and maxLAVi.

In the early nineties, Appleton et al. [7] noticed that a minLAV of ≥40 mL predicted a pulmonary wedge pressure >12 mmHg with a sensitivity and specificity of 82 and 98 %, respectively. Until recently, minLAV was almost ignored. The left atrial function has traditionally been described as modulating LV filling in three phases: the reservoir phase (during atrial relaxation and LV systole, ending up in maxLAV), and the conduit and the atrial contraction phases (during LV filling, ending up in minLAV). As opposed to that observed during the reservoir phase, during the conduit and atrial contraction phases, the LA is directly exposed to LV pressure. An increase in LV filling pressure may directly affect LA pressure and, therefore, LA size. Consequently, minLAV may be a more sensitive marker of an increased LV filling pressure than maxLAV. Supportive of this hypothesis, Russo et al. [29] recently found a considerably stronger association between E/e′ ratio and minLAV than between E/e′ ratio and maxLAV.

The present study confirmed previous findings that minLAVi increase with age [28, 30]; however, we did not assess the pre-atrial contraction volume and may, therefore, only speculate on this age dependency. In contrast with the LA reservoir function, previous studies have shown that the conduit function deteriorates with age [28, 30]. The observed decrease in passive atrial emptying is most likely related to changed LV filling properties associated with age. However, the LA contraction function seems to be maintained or even amplified with age [28, 30]. The observed increase in LA active pump function with age may be a compensation for the impaired early filling and is possibly mediated by the Frank-Starling mechanism. However, the fact that our data and those of others showed a rise in minLAVi with age suggests that increased atrial contractility may not fully compensate for the age related decrease in passive LA emptying.

The intra- and interobserver reproducibility of the LA volume measurements in the present study were very similar to the recent findings of Aune et al. [19], including a slightly poorer reproducibility for minLAV compared with maxLAV. As the LA is at its smallest size at minLAV, the echo broadening in atrial septum increases because of an augmentation in atrial septal thickness, rendering it harder to identify the true wall echoes, which may explain the difference in reproducibility observed between minLAV and maxLAV.

Because LA enlargement is observed frequently in subjects with LV diastolic dysfunction, a condition that presents frequently in elderly individuals, we made an effort to exclude the possibility of a systematic bias, i.e., that several of the participants had preclinical diastolic dysfunction. Therefore, conventional Doppler indices, tissue Doppler filling indices, and NT-proBNP levels were analyzed. The distributions of diastolic Doppler measurements in the present study population were within what could be expected in a population without apparent cardiovascular disease compared with findings from a large Scandinavian study of healthy subjects [31]. In addition, based on an EACVI-guideline-recommended algorithm [13], an analysis suggested a low burden of possible preclinical diastolic dysfunction in the present study population.

## Limitations and strengths

The present population-based subset of men and women were all of northern European descent; therefore, the extent to which the data can be extrapolated to other ethnic groups is not known. Only 41 (24 %) of the participants were women, which may have reduced the statistical power. The main reason for the sex difference was that, for each patient included in the VaMIS study, a control subject matched for age and sex was recruited from the general population. Consequently, the sex distribution of the control subjects who were enrolled in the present study reflects the sex difference in patients hospitalized for myocardial infarction.

Unfortunately, the acquisition and storage of loops dedicated to LA planimetry were only obtained in the 4-chamber view, and not in the 2-chamber view in the present study. Thus, the guideline-recommended biplane assessment of LA volumes [8] was not possible, which represented a limitation of the study. However, biplane planimetry is quite often unfeasible because of suboptimal image quality in the apical 2-chamber view, preventing adequate visualization of the LA anterior wall [21]. Lester et al. [32] demonstrated that the mean ± SD of the absolute difference between the single-plane and biplane disc methods was 6 ± 5 mL, indicating a strong agreement between the two methods. Although bi- and single-plane assessment of LA volumes are not interchangeable, previous studies have suggested that the single-plane method may be acceptable for clinical use if reference limits that are specific for the method are available [21, 32].

The intra- and interobserver variability was obtained from the same set of images, i.e., only the measurement variability was tested, and not inconsistencies caused by variations in imaging planes or beat-to-beat variations.

The strengths of the present study included the population-based design and the well-characterized participants without signs of cardiovascular disease or risk factors and who were not taking any medications. The evaluation of LV diastolic function and NT-proBNP concentration was important considering the well-known association between LV filling function and LA volumes [13].

## Conclusions

In the present population-based random sample of middle-aged and older subjects without known or apparent cardiovascular disease or comorbidity, the maximum and minimum LA volumes were studied using the single-plane disc method. The current data confirmed that maxLAVi is independent of age and sex, and showed that it displayed an upper normal limit of 37 mL/m^2^. In contrast, minLAVi was correlated with age and exhibited estimated upper reference limits of 15 and 23 mL/m^2^ in subjects aged 40 and 80 years, respectively.
